# Bridging Nerve Defects

**Published:** 1904-12-10

**Authors:** 


### BRIDGING NERVE DEFECTS.

The successes which have followed simple nerve suture are now matters of common knowledge and no surgeon would hesitate to unite the two ends of a divided nerve with a feeling of assurance that, provided the wound healed in a healthy manner, the ultimate functional result would be a good one. But what is he to do if, from actual loss of substance or for other reason, the two ends cannot be approximated, even by stretching the proximal and distal portions of the nerve? That is a question which has perplexed more than one operator at an awkward moment. As is so usual where no definite line of treatment is certain to produce the required effect, the text-books give a large number of alternative methods from which the practitioner may select according to his fancy. Thus he is advised to graft a piece of nerve removed from a dog or rabbit or some other animal into the gap, or else to perform a flap operation, or to implant the distal nerve section into an uninjured neighbouring nerve trunk, or he may shorten a limb by resection of a piece of bone, so that the nerve ends may be brought together, or he may simply connect the two ends with catgut in the hope that new nerve tissue may grow along the track of the catgut, and if he chooses he may wrap a piece of tissue round the catgut ligatures to prevent the surrounding tissues from obliterating the tract. Dr. C. A. Powers has endeavoured by scrutinising the literature to discover which of these several methods has been attended with the largest measure of success. The result of his inquiry, which is published in the current number of the "Annals of Surgery," is rather discouraging. He shows that a number of the cases reported as being successful have been published too soon after the operation to be of any value, and it is possible that in some of these the early return of sensation may be explained by collateral nerve supply, and early return of motion may be attributed to vicarious movements of other muscles than those formerly paralysed. He concludes that no definite method can be advocated until more cases, and especially cases which are more carefully recorded, are available for analysis. At present it seems that neuroplasty and implantation are the two methods of choice, and that resection of bone may be advisable in selected cases, while the other methods, including nerve grafting, must be abandoned as useless.

